# The refinement and generalization of Hardy’s inequality in Sobolev space

**DOI:** 10.1186/s13660-018-1922-5

**Published:** 2018-11-28

**Authors:** Xiaomin Xue, Fushan Li

**Affiliations:** 1 School of Mathematical Sciences, Qufu Normal University, Qufu, P.R. China; 2 Center for Teaching Research and Evaluation, Qufu Normal University, Qufu, P.R. China

**Keywords:** Hardy’s inequality, Integrate by part, Cauchy inequality, Divergence theorem

## Abstract

In this paper, we refine the proof of Hardy’s inequality in (Evans in Partial Differential Equations, 2010, Hardy in Inequalities, 1952) and extend Hardy’s inequality from two aspects. That is to say, we extend the integral estimation function from $\frac{u}{|x|}$ to $\frac{u}{|x|^{\sigma }}$ with suitable $\sigma >0$ and extend the space dimension from $n\geq 3$ to $n\geq 2$. Hardy’s inequality in (Evans in Partial Differential Equations, 2010, Hardy in Inequalities, 1952) is the special case of our results.

## Introduction

It is well known that inequalities are important tools in classical analysis [2–6, 13, 14, 26–29, 31–39, 41–43, 45]. One application of inequalities is to study the properties of partial differential equations. Li and his coauthors [15–23] studied the global existence and uniqueness, limit behavior, uniform stability, and blow-up of solutions for partial differential equations by using various inequalities. Liu [11, 24, 25] showed the stability and convergence results of evolution equations and Du [8, 9] studied obstacle problems by using various inequalities.

In recent decades, there have been many results on the extension and refinement of inequality [7, 10, 12, 30, 40, 44]. Qin [30] summarized a large number of inequalities and applications, but Hardy’s inequality was not included. The authors [7, 40] generalized the summation form Hardy inequality, Zhang [44] extended Hardy inequalities using Littlewood–Paley theory and nonlinear estimates method in Besov spaces, and the results improved and extended the well-known results in [1].

The first edition of classic textbook [10] does not contain Hardy’s inequality, we see that the very significant Hardy’s inequality

$$ \int _{B(o,r)}\frac{u^{2}}{ \vert x \vert ^{2}}\,dx \leq C \int _{B(o,r)} \biggl( \vert Du \vert ^{2}+ \frac{u ^{2}}{r^{2}} \biggr)\,dx $$

holds if $u\in H^{1}(B(o,r))$, $n\geq 3$, and $r>0$ in the second edition of [10]. The proof of Hardy’s inequality given in [10, 12] is very ingenious, but it is not easy to master for the reader. Therefore, we refine the proof of Hardy’s inequality for readers to grasp the essence of the proof and extend Hardy’s inequality in Sobolev space from two aspects. That is to say, we extend the integral estimation function from $\frac{u}{|x|}$ to $\frac{u}{|x|^{ \sigma }}$ with suitable $\sigma >0$ and extend the space dimension from $n \geq 3$ to $n \geq 2$. Hardy’s inequality in [10, 12] is the special case of our results.

Let $B(o,r)$ be a closed ball in $\mathbf{R}^{n}$ with center *o* and radius $r>0$, $x=(x_{1},x_{2},\ldots , x_{n})$ be a vector in $B(o,r)$, $\nu =(\nu _{1},\nu _{2},\ldots ,\nu _{n})= (\frac{x_{1}}{r},\frac{x _{2}}{r},\ldots ,\frac{x_{n}}{r} )$ be the unit outward normal to $\partial B(o,r)$. $W^{k,p}(\varOmega )$ and $H^{1}(\varOmega )$ denote the Sobolev spaces. We write

$$\begin{aligned} Du=(u_{x_{1}},u_{x_{2}},\ldots , u_{x_{n}}), \quad \vert x \vert = \bigl(x_{1}^{2}+x_{2}^{2}+ \cdots +x_{n}^{2} \bigr)^{\frac{1}{2}}. \end{aligned}$$

In Sect. 2, we first recall Hardy’s inequality, refine the proof for completeness, and state our main results. The proofs of the main results are given in Sect. 3.

## Main results

Now, we present the global approximation theorem and Hardy’s inequality in Sobolev space.

### Lemma 2.1

([10], Global approximation theorem)

*Assume that* *Ω* *is bounded and* *∂Ω* *is* $C^{1}$. *Let* $u\in W^{k,p}(\varOmega )$ *for some* $1\leq p<\infty $. *Then there exist functions* $u_{m}\in C^{\infty }(\overline{\varOmega })$ *such that*

$$\begin{aligned} u_{m}\rightarrow u \quad \mathit{in } W^{k,p}(\varOmega ). \end{aligned}$$

### Lemma 2.2

([10, 12], Hardy’s inequality)

*Assume* $n\geq 3$ *and* $r>0$. *Let* $u\in H^{1}(B(o,r))$. *Then* $\frac{u}{|x|} \in L^{2}(B(o,r))$ *with the estimate*

2.1 $$\begin{aligned} \int _{B(o,r)}\frac{u^{2}}{ \vert x \vert ^{2}}\,dx \leq C \int _{B(o,r)} \biggl( \vert Du \vert ^{2}+ \frac{u ^{2}}{r^{2}} \biggr)\,dx. \end{aligned}$$

For readers to grasp the essence of the proof, we give the refined proof below.

### Proof

By the global approximation theorem Lemma 2.1, we may assume $u\in C^{\infty }(B(o,r))$. Noting that $D (\frac{1}{|x|^{ \rho }} )=-\rho \frac{x}{|x|^{\rho +2}}$ for any $\rho >0$ and integrating by parts, we have

2.2 $$\begin{aligned} \int _{B(o,r)}\frac{u^{2}}{ \vert x \vert ^{2}}\,dx &= -\frac{1}{\rho } \int _{B(o,r)}u ^{2}D \biggl(\frac{1}{ \vert x \vert ^{\rho }} \biggr) \cdot \frac{x}{ \vert x \vert ^{2-\rho }}\,dx \\ &= -\frac{1}{\rho } \int _{B(o,r)}u^{2}\sum_{i=1}^{n} \biggl(\frac{1}{ \vert x \vert ^{ \rho }} \biggr)_{x_{i}} \frac{x_{i}}{ \vert x \vert ^{2-\rho }}\,dx \\ &= -\frac{1}{\rho } \int _{\partial B(o,r)}\sum_{i=1}^{n}u^{2} \nu _{i} \cdot \frac{x_{i}}{ \vert x \vert ^{2}}\,dS \\ &\quad {}+\frac{1}{\rho } \int _{B(o,r)}\sum_{i=1}^{n} \frac{1}{ \vert x \vert ^{\rho }} \biggl(u^{2}\frac{x_{i}}{ \vert x \vert ^{2-\rho }} \biggr)_{x_{i}} \,dx \\ &= -\frac{1}{\rho r} \int _{\partial B(o,r)}u^{2}\,dS \\ &\quad {}+\frac{1}{\rho } \int _{B(o,r)} \biggl[2uDu\cdot \frac{x}{ \vert x \vert ^{2}}+(n+ \rho -2) \frac{u^{2}}{ \vert x \vert ^{2}} \biggr]\,dx. \end{aligned}$$

Therefore

2.3 $$\begin{aligned} (n-2) \int _{B(o,r)}\frac{u^{2}}{ \vert x \vert ^{2}}\,dx =-2 \int _{B(o,r)}uDu\cdot \frac{x}{ \vert x \vert ^{2}}\,dx+\frac{1}{r} \int _{\partial B(o,r)}u^{2}\,dS. \end{aligned}$$

For any $\varepsilon >0$, using the Cauchy inequality and Schwarz inequality, we obtain

$$\begin{aligned} -2 \int _{B(o,r)}uDu\cdot \frac{x}{ \vert x \vert ^{2}}\,dx &= -2 \int _{B(o,r)} \frac{u}{ \vert x \vert }Du\cdot \frac{x}{ \vert x \vert }\,dx \\ &\leq 2 \int _{B(o,r)}\frac{ \vert u \vert }{ \vert x \vert } \vert Du \vert \biggl\vert \frac{x}{ \vert x \vert } \biggr\vert \,dx \\ &= 2 \int _{B(o,r)}\frac{ \vert u \vert }{ \vert x \vert } \vert Du \vert \,dx \\ &\leq 2 \varepsilon \int _{B(o,r)}\frac{u^{2}}{ \vert x \vert ^{2}}\,dx+\frac{1}{2 \varepsilon } \int _{B(o,r)} \vert Du \vert ^{2}\,dx. \end{aligned}$$

Fixing $\varepsilon >0$ such that $n-2-2\varepsilon >0$, we conclude

2.4 $$\begin{aligned} \int _{B(o,r)}\frac{u^{2}}{ \vert x \vert ^{2}}\,dx \leq C \int _{B(o,r)} \vert Du \vert ^{2}\,dx+ \frac{C}{r} \int _{\partial B(o,r)}u^{2}\,dS. \end{aligned}$$

According to the divergence theorem, we have

2.5 $$\begin{aligned} \int _{B(o,r)}\operatorname{div}\bigl(xu^{2}\bigr)\,dx &= \int _{\partial B(o,r)}xu^{2} \cdot \nu \,dS= \int _{\partial B(o,r)}u^{2}x\cdot \frac{x}{r}\,dS \\ &=r \int _{\partial B(o,r)}u^{2}\,dS. \end{aligned}$$

Using the Cauchy inequality and Schwarz inequality, we get

2.6 $$\begin{aligned} \int _{B(o,r)}\operatorname{div}\bigl(xu^{2}\bigr)\,dx = & \int _{B(o,r)} \bigl[u^{2} \operatorname{div}(x)+D \bigl(u^{2}\bigr)\cdot x \bigr]\,dx \\ = & \int _{B(o,r)} \bigl(nu^{2}+2uDu\cdot x \bigr)\,dx \\ \leq & \int _{B(o,r)} \bigl(nu^{2}+u^{2}+ \vert x \vert ^{2} \vert Du \vert ^{2} \bigr)\,dx \\ \leq & \int _{B(o,r)} \bigl[(n+1)u^{2}+r^{2} \vert Du \vert ^{2} \bigr]\,dx. \end{aligned}$$

Combining (2.5) and (2.6), we obtain the trace inequality

2.7 $$\begin{aligned} \frac{1}{r} \int _{\partial B(o,r)}u^{2}\,dS \leq C \int _{B(o,r)} \biggl( \vert Du \vert ^{2}+ \frac{u ^{2}}{r^{2}} \biggr)\,dx. \end{aligned}$$

Employing this inequality (2.7) in (2.4) finishes the proof of (2.1). □

Under the circumstance, we extend the space dimension *n* and parameter *σ* in $\frac{u}{|x|^{\sigma }}$ of Hardy’s inequality. Now we show our main results.

### Theorem 2.1

*Assume* $n\geq 2$ *and* $r>0$, $u\in H^{1}(B(o,r))$. *Then*, *for* $\sigma <\frac{n}{2}$, *we have* $\frac{u}{|x|^{\sigma }}\in L^{2}(B(o,r))$ *with the estimate as follows*:

*If* $\sigma \leq 1$ *and* $\sigma < \frac{n}{2}$, *we have*

$$\begin{aligned} \int _{B(o,r)}\frac{u^{2}}{ \vert x \vert ^{2\sigma }}\,dx \leq C \int _{B(o,r)} \biggl(\frac{ \vert Du \vert ^{2}}{r ^{2(\sigma -1)}} +\frac{u^{2}}{r^{2\sigma }} \biggr)\,dx. \end{aligned}$$

*If* $\sigma >1$ *and* $\sigma < \frac{n}{2}$, *we have*

$$\begin{aligned} \int _{B(o,r)}\frac{u^{2}}{ \vert x \vert ^{2\sigma }}\,dx\leq C \int _{B(o,r)} \biggl(\frac{ \vert Du \vert ^{2}}{ \vert x \vert ^{2( \sigma -1)}} +\frac{u^{2}}{r^{2\sigma }} \biggr)\,dx. \end{aligned}$$

### Remark 2.1

Hardy’s inequality (2.1) is the case of $\sigma =1$ and $n\geq 3$ in Theorem 2.1.

### Remark 2.2

If $n=2$, then $\sigma <1$. $B(o,r)$ denotes a closed circular region with center *o* and radius $r>0$, $\partial B(o,r)$ denotes a circle, and $\int _{B(o,r)}\cdots dS$ denotes curvilinear integration.

## Proofs of the main results

In this section we show the proofs of the main results Theorem 2.1.

### Proof

For any $\rho >0$, since

$$\begin{aligned} D \biggl(\frac{1}{ \vert x \vert ^{\rho }} \biggr)=-\rho \frac{x}{ \vert x \vert ^{\rho +2}}, \end{aligned}$$

which implies

3.1 $$\begin{aligned} \frac{1}{{ \vert x \vert }^{2\sigma }} &= \biggl[-\rho \frac{x}{{ \vert x \vert }^{\rho +2}} \biggr] \cdot \biggl[ \biggl(-\frac{1}{ \rho } \biggr)\frac{x}{{ \vert x \vert }^{2\sigma -\rho }} \biggr] \\ &=-\frac{1}{\rho }D \biggl(\frac{1}{{ \vert x \vert }^{\rho }} \biggr)\cdot \frac{x}{ { \vert x \vert }^{2\sigma -\rho }} . \end{aligned}$$

By the global approximation theorem, we may assume $u\in C^{\infty }(B(o,r))$. Noting that (3.1) holds, we obtain

3.2 $$\begin{aligned} \int _{B(o,r)}\frac{u^{2}}{ \vert x \vert ^{2\sigma }}\,dx &= -\frac{1}{\rho } \int _{B(o,r)}u^{2}D \biggl(\frac{1}{ \vert x \vert ^{\rho }} \biggr) \cdot \frac{x}{ \vert x \vert ^{2 \sigma -\rho }}\,dx \\ &= -\frac{1}{\rho } \int _{B(o,r)}\sum_{i=1}^{n} \biggl(\frac{1}{ \vert x \vert ^{ \rho }} \biggr)_{x_{i}} \biggl(u^{2} \frac{x_{i}}{ \vert x \vert ^{2\sigma -\rho }} \biggr)\,dx \\ &= -\frac{1}{\rho } \int _{\partial B(o,r)}\sum_{i=1}^{n}u^{2} \nu _{i} \cdot \frac{x_{i}}{ \vert x \vert ^{2\sigma }}\,dS \\ &\quad {}+\frac{1}{\rho } \int _{B(o,r)}\sum_{i=1}^{n} \frac{1}{ \vert x \vert ^{\rho }} \biggl(u^{2}\frac{x_{i}}{ \vert x \vert ^{2\sigma -\rho }} \biggr)_{x_{i}} \,dx \\ &= -\frac{1}{\rho r^{2\sigma -1}} \int _{\partial B(o,r)}u^{2}\,dS \\ &\quad {}+\frac{1}{\rho } \int _{B(o,r)} \biggl[2uDu\cdot \frac{x}{ \vert x \vert ^{2\sigma }}+(n+\rho -2\sigma )\frac{u^{2}}{ \vert x \vert ^{2\sigma }} \biggr]\,dx. \end{aligned}$$

Hence

3.3 $$\begin{aligned} (n-2\sigma ) \int _{B(o,r)}\frac{u^{2}}{ \vert x \vert ^{2\sigma }}\,dx &= -2 \int _{B(o,r)}uDu \cdot \frac{x}{ \vert x \vert ^{2\sigma }}\,dx \\ &\quad {}+\frac{1}{r^{2\sigma -1}} \int _{\partial B(o,r)}u^{2}\,dS. \end{aligned}$$

For any $\varepsilon >0$, using the Cauchy inequality and Schwarz inequality, we obtain

3.4 $$\begin{aligned} -2 \int _{B(o,r)}uDu\cdot \frac{x}{ \vert x \vert ^{2\sigma }}\,dx &= -2 \int _{B(o,r)}\frac{u}{ \vert x \vert ^{ \sigma }}Du\cdot \frac{x}{ \vert x \vert ^{\sigma }}\,dx \\ &\leq 2 \int _{B(o,r)}\frac{ \vert u \vert }{ \vert x \vert ^{\sigma }} \vert Du \vert \biggl\vert \frac{x}{ \vert x \vert ^{ \sigma }} \biggr\vert \,dx \\ &= 2 \int _{B(o,r)}\frac{ \vert u \vert }{ \vert x \vert ^{\sigma }} \vert Du \vert \frac{1}{ \vert x \vert ^{\sigma -1}}\,dx \\ &\leq 2\varepsilon \int _{B(o,r)}\frac{u^{2}}{ \vert x \vert ^{2\sigma }}\,dx \\ &\quad {}+\frac{1}{2\varepsilon } \int _{B(o,r)} \vert Du \vert ^{2}\frac{1}{ \vert x \vert ^{2(\sigma -1)}} \,dx. \end{aligned}$$

According to the divergence theorem, we have

$$\begin{aligned} \int _{B(o,r)}\operatorname{div}\bigl(xu^{2}\bigr)\,dx=r \int _{\partial B(o,r)}u^{2}\,dS, \end{aligned}$$

and using the Cauchy inequality and Schwarz inequality, we get

$$\begin{aligned} \int _{B(o,r)}\operatorname{div}\bigl(xu^{2}\bigr)\,dx &= \int _{B(o,r)} \bigl(nu^{2}+2uDu \cdot x \bigr)\,dx \\ &\leq \int _{B(o,r)} \bigl(nu^{2}+u^{2}+ \vert Du \vert ^{2} \vert x \vert ^{2} \bigr)\,dx \\ &\leq \int _{B(o,r)} \bigl[(n+1)u^{2}+r^{2} \vert Du \vert ^{2} \bigr]\,dx, \end{aligned}$$

which implies

3.5 $$\begin{aligned} \frac{1}{r^{2\sigma -1}} \int _{\partial B(o,r)}u^{2}\,dS \leq \frac{n+1}{r ^{2\sigma }} \int _{B(o,r)}u^{2}\,dx+\frac{1}{r^{2(\sigma -1)}} \int _{B(o,r)} \vert Du \vert ^{2}\,dx. \end{aligned}$$

By substituting (3.4) and (3.5) into (3.3), fixing *ε* such that $n-2\sigma -2\varepsilon >0$, we conclude

3.6 $$\begin{aligned} \int _{B(o,r)}\frac{u^{2}}{ \vert x \vert ^{2\sigma }}\,dx \leq C \int _{B(o,r)} \biggl[\frac{ \vert Du \vert ^{2}}{ \vert x \vert ^{2( \sigma -1)}} +\frac{ \vert Du \vert ^{2}}{r^{2(\sigma -1)}}+ \frac{u^{2}}{r^{2 \sigma }} \biggr]\,dx. \end{aligned}$$

Therefore, from (3.6), for $n\geq 2$ and $\sigma <\frac{n}{2}$:

if $\sigma \leq 1$, noting that

$$\begin{aligned} \frac{ \vert Du \vert ^{2}}{ \vert x \vert ^{2(\sigma -1)}}\leq \frac{ \vert Du \vert ^{2}}{r^{2(\sigma -1)}}, \quad x\in B(o,r), \end{aligned}$$

we obtain

3.7 $$\begin{aligned} \int _{B(o,r)}\frac{u^{2}}{ \vert x \vert ^{2\sigma }}\,dx \leq C \int _{B(o,r)} \biggl(\frac{ \vert Du \vert ^{2}}{r ^{2(\sigma -1)}}+\frac{u^{2}}{r^{2\sigma }} \biggr) \,dx. \end{aligned}$$

if $\sigma >1$, noting that

$$\begin{aligned} \frac{ \vert Du \vert ^{2}}{ \vert x \vert ^{2(\sigma -1)}}\geq \frac{ \vert Du \vert ^{2}}{r^{2(\sigma -1)}}, \quad x\in B(o,r), \end{aligned}$$

we obtain

3.8 $$\begin{aligned} \int _{B(o,r)}\frac{u^{2}}{ \vert x \vert ^{2\sigma }}\,dx \leq C \int _{B(o,r)} \biggl(\frac{ \vert Du \vert ^{2}}{ \vert x \vert ^{2( \sigma -1)}} +\frac{u^{2}}{r^{2\sigma }} \biggr)\,dx. \end{aligned}$$

The proof of Theorem 2.1 is completed. □

## Conclusions

In this paper, we refine the proof of Hardy’s inequality for readers to grasp the essence of the proof and extend Hardy’s inequality in Sobolev space from two aspects. That is to say, we extend the integral estimation function from $\frac{u}{|x|}$ to $\frac{u}{|x|^{\sigma }}$ with suitable $\sigma >0$ and extend the space dimension from $n \geq 3$ to $n \geq 2$. Hardy’s inequality in [10, 12] is the special case of our results.
